# Gene mutations of platelet glycoproteins and response to tirofiban in acute coronary syndrome: Mutações gênicas das glicoproteínas plaquetárias e resposta ao tirofiban na síndrome coronariana aguda

**DOI:** 10.1590/1516-3180.2015.00650808

**Published:** 2016-01-19

**Authors:** Antonio de Padua Mansur, Alessandra Roggerio, Júlio Yoshio Takada, Pérola Michelle Vasconcelos Caribé, Solange Desirée Avakian, Célia Maria Cassaro Strunz

**Affiliations:** I MD, PhD. Associate Professor, Department of Cardiopulmonology, Instituto do Coração, Hospital das Clínicas, Faculdade de Medicina, Universidade de São Paulo, São Paulo, Brazil.; II BSc, PhD. Biochemist, Department of Cardiopulmonology, Instituto do Coração, Hospital das Clínicas, Faculdade de Medicina, Universidade de São Paulo, São Paulo, Brazil.; III MD, PhD. Attending Physician, Department of Cardiopulmonology, Instituto do Coração, Hospital das Clínicas, Faculdade de Medicina, Universidade de São Paulo, São Paulo, Brazil.; IV MD, MSc. Doctoral Student, Department of Cardiopulmonology, Instituto do Coração, Hospital das Clínicas, Faculdade de Medicina, Universidade de São Paulo, São Paulo, Brazil.; V BSc. Central Laboratory Director, Department of Cardiopulmonology, Instituto do Coração, Hospital das Clínicas, Faculdade de Medicina, Universidade de São Paulo, São Paulo, Brazil.

**Keywords:** Glycoproteins, Platelet glycoprotein GPIIb-IIIa complex, Polymorphism, genetic, Acute coronary syndrome, Angina, unstable, Myocardial infarction

## Abstract

**CONTEXT AND OBJECTIVES:**

Glycoprotein inhibitors (abciximab, eptifibatide and tirofiban) are used in patients with unstable angina and non-ST-segment elevation myocardial infarction before percutaneous coronary intervention. Of these, tirofiban is the least effective. We hypothesized that the response to tirofiban might be associated with glycoprotein gene mutations.

**DESIGN AND SETTING:**

Prospective study at Emergency Unit, Heart Institute (InCor), University of São Paulo.

**METHOD:**

Intrahospital evolution and platelet aggregation in response to tirofiban were analyzed in relation to four glycoprotein mutations in 50 patients indicated for percutaneous coronary intervention: 17 (34%) with unstable angina and 33 (66%) with non-ST-segment elevation myocardial infarction. Platelet aggregation was analyzed using the Born method. Blood samples were obtained before and one hour after tirofiban infusion. Glycoproteins Ia (*807C/T*), Ib *(Thr/Met)*, IIb (*Ile/Ser*) and IIIa (*PIA*) were the mutations selected.

**RESULTS:**

Hypertension, dyslipidemia, diabetes, smoking, previous coronary artery disease and stroke were similar between the groups. Mutant glycoprotein IIIa genotypes had lower platelet aggregation before tirofiban administration than that of the wild genotype (41.0% ± 22.1% versus 55.9% ± 20.8%; P = 0.035). Mutant glycoprotein IIIa genotypes correlated moderately with lower platelet inhibition (r = -0.31; P = 0.030). After tirofiban administration, platelet glycoprotein Ia, Ib, IIb and IIIa mutations did not influence the degree of inhibition of platelet aggregation or intrahospital mortality.

**CONCLUSIONS:**

Mutations of glycoproteins Ia, Ib, IIb and IIIa did not influence platelet aggregation in response to tirofiban in patients with unstable angina and non-ST-segment elevation myocardial infarction.

## RESUMO

**CONTEXTO E OBJETIVOS:**

Inibidores da glicoproteína (abciximab, eptifibatide, tirofiban) são utilizados em pacientes com angina instável e infarto do miocárdio sem elevação do segmento ST (IAMSSST) antes da intervenção coronária percutânea. Dentre eles, o tirofiban é o menos eficaz. Nossa hipótese é que a resposta ao tirofiban possa estar associada a mutações no gene da glicoproteína.

**DESENHO E LOCAL:**

Estudo prospectivo na Unidade de Emergência do Instituto do Coração (InCor), Universidade de São Paulo (USP).

**MÉTODOS:**

Foram analisadas a evolução intra-hospitalar e agregabilidade plaquetária em resposta ao tirofiban de 4 mutações da glicoproteína em 50 pacientes com indicação para intervenção coronária percutânea, 17 (34%) com angina instável e 33 (66%) com IAMSSST. A agregação plaquetária foi analisada pelo método de Born. Amostras de sangue foram obtidas antes e uma hora após infusão do tirofiban. As glicoproteínas Ia (*807C/T*), Ib (*Thr/Met*), IIb (*Ile/Ser*) e IIIa (*PIA*) foram as mutações selecionadas.

**RESULTADOS:**

Hipertensão, dislipidemia, diabetes, tabagismo, doença coronariana e acidente vascular cerebral prévios foram semelhantes entre os grupos. Observou-se menor agregabilidade plaquetária dos genótipos mutantes da glicoproteína IIIa antes da administração de tirofiban do genótipo selvagem (41% ± 22% *versus* 56% ± 21%; P = 0,035). Genótipos mutantes da glicoproteína IIIa correlacionaram-se moderadamente com menor inibição plaquetária (r = -0,31; P = 0,030). Após a administração tirofiban, as mutações das glicoproteínas Ia, Ib, IIb, e IIIa não influenciaram o grau de inibição da agregação plaquetária e mortalidade intra-hospitalar.

**CONCLUSÕES:**

Mutações das glicoproteínas Ia, Ib, IIb e IIIa não influenciaram a agregação plaquetária em resposta ao tirofiban nos pacientes com angina instável e IAMSSST.

## INTRODUCTION

Tirofiban, a platelet surface receptor glycoprotein IIbIIIa (GPIIbIIIa) inhibitor, is recommended for patients with unstable angina and non-ST-segment elevation myocardial infarction (NSTEMI) before a planned percutaneous coronary intervention, in order to reduce periprocedural coronary events. GPIIbIIIa plays a central role in thrombus formation through binding fibrinogen, von Willebrand factor and fibronectin.^1^ Tirofiban, like fibrinogen, has a similar affinity to the active GPIIbIIIa receptor. Thus, tirofiban binds to GPIIbIIIa and prevents formation of fibrinogen bridges, thereby reducing the thrombogenesis process.^2^ Clinical studies have demonstrated the efficacy of tirofiban in reducing coronary events in patients with unstable angina and NSTEMI who undergo percutaneous coronary intervention.^3^^,^^4^ However, studies have shown unfavorable results from use of tirofiban for reducing ischemic events in patients undergoing percutaneous coronary intervention and also less inhibition of platelet aggregation with tirofiban, compared with two other commercially available inhibitors of platelet glycoprotein, abciximab and eptifibatide.^5^^,^^6^ The possibility of a paradoxical effect of platelet inhibitors on platelet aggregation has also been discussed.^7^ The possible hypotheses for these findings relate to lower intensity of platelet inhibition, antagonist-induced platelet activation and interaction between platelets and inflammation.^8^ Another hypothesis to explain the interindividual variability in the antiplatelet effect of tirofiban may involve platelet glycoprotein gene polymorphisms.^9^ Mutations of GPIa (*807C/T*), Ib (*Thr/Met*), IIb (*Ile/Ser*) and IIIa (*PIA*) have been inconsistently associated with increased risk of major acute coronary events and resistance to antiplatelet drugs.^10^^,^^11^ We hypothesized that the smaller effect of tirofiban might be associated with platelet GP gene mutations.

## METHODS

Baseline clinical and admission laboratory characteristics, coronary artery disease risk factors, in-hospital outcomes, angiography and treatments were analyzed in relation to 50 consecutive patients admitted to the emergency room with unstable angina or NSTEMI between May 2008 and November 2010. They were indicated to receive tirofiban before undergoing coronary angiography for possible percutaneous coronary intervention, as advocated in the ACC/AHA 2002 guidelines for management of patients with unstable angina and non-ST-segment elevation myocardial infarction.^12^

All the patients received 200 mg of aspirin or were put on aspirin at least 3 hours before tirofiban was started, along with heparin. Initially, a bolus dosage regimen of 0.4 µg/kg/min was given for 30 minutes, followed by a maintenance dose of 0.1 µg/kg/min for at least 24 hours.

High-risk unstable angina was defined as chest pain at rest lasting for more than 20 minutes, with dynamic electrocardiographic changes on the electrocardiogram and normal levels of MB isoenzyme of creatine kinase (CKMB) mass or troponin I. NSTEMI was diagnosed when these findings were associated with increased blood levels of CKMB and troponin I. The clinical outcome was defined as in-hospital death from all causes. The study was approved by the local institutional ethics committee. Informed consent was obtained from all patients.

### Laboratory analysis

Troponin I and CKMB mass levels were analyzed in serum samples using specific kits, in the automated ADVIA Centaur equipment (Siemens Healthcare Diagnostics, Tarrytown, NY, USA). The kit used for troponin determination was TnI-Ultra, and all assays were conducted in accordance with the manufacturer’s instructions. Platelet aggregation tests in vitro were analyzed before and one hour after tirofiban administration. Venous blood was collected in citrated tubes before and one hour after the introduction of tirofiban. Plasma samples were obtained after centrifugation and were analyzed as soon as possible. Platelet-rich plasma (PRP) was obtained by means of centrifugation at 250 g for 4 minutes from citrated blood. The time between sample collection and the tests did not exceed two hours. The platelet aggregation was performed by means of the optical method and consisted of treating 400 ul of PRP with 10 uM ADP, in the Chrono-Log 440 equipment (Havertown, PA, USA) in accordance with the Born method.^13^ The mixture was stirred at 37 °C using Teflon-coated magnetic rods, and aggregation curves were recorded using an aggregometer. The analysis was performed in duplicate, and the aggregation rate was measured from the maximum variation of light transmittance from the system.

### Platelet glycoprotein genotype determination

Samples for DNA extraction were collected in EDTA tubes and maintained at 4 °C until use. Genomic DNA was isolated from peripheral blood lymphocytes in accordance with the method of Miller et al.^14^ Platelet membrane glycoprotein Ia, Ibα, IIb and IIIa genotypes were analyzed by means of amplification of DNA using the polymerase chain reaction (PCR), as described in previously published methods,^15^^,^^16^^,^^17^ and using the oligonucleotide sequencing primers described in Table 1. The mutations analyzed were selected because these mutations present high frequency in our population.^11^

**Table 1.**
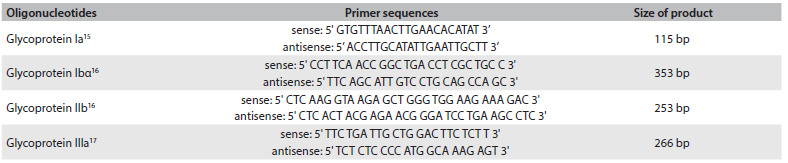
Primer sequences and expected sizes of the products in base pairs (BP)

Genotyping of glycoprotein Ia (*807C/T*) polymorphism was performed by means of enzymatic digestion of a 115 bp fragment using Taq I at 37 °C, overnight, followed by identification of the fragments by means of electrophoresis on 2.5% agarose gel.^15^ Presence of the 92 bp fragment indicates homozygosis of the wild genotype (C/C), while presence of the 115 bp fragment indicates homozygosis of the mutant allele.

Presence of the platelet glycoprotein Ibα (*Thr/Met*) genotype characterizes substitution of a cytosine by a thymine at position 1018 and results in amino acid dimorphism (*Thr/Met*) in the 145 position of GP Ibα. A 353 bp sequence of nucleotides^16^ resulting from PCR was subjected to digestion using BsaHI at 37 °C overnight, followed by identification of fragments through electrophoresis.

The normal allele has a restriction site for BsaHI, thus resulting in two fragments of 242 and 111 bp. Presence of the mutant allele leads to loss of the restriction site (353 bp fragment).

Presence of the platelet glycoprotein IIb (*Ile/Ser*) genotype was reflected in Ile/Ser dimorphism,^16^ which occurred at position 843 of GPIIb, thus resulting in substitution of thymine for guanine at nucleotide 2622 in exon 26. A 253 bp sequence of nucleotides was amplified using PCR and was digested with Fok I at 37 °C overnight; the fragments were identified by means of electrophoresis.

The normal allele had a restriction site for Fok I, thus resulting in two fragments of 127 and 111 bp. Presence of the mutant allele leads to loss of the restriction site and a fragment of 253 bp.

Presence of the platelet glycoprotein IIIa (*PIA*) genotype resulted from the C/T transition at position 1565 in exon 2 of GP IIIa.^17^ The PCR reaction product, comprising a sequence of 266 bp of nucleotides was digested using MspI, at 37 °C overnight, followed by identification of fragments by means of electrophoresis. The normal allele had a restriction site for MspI, thus resulting in two fragments of 221 and 45 bp. The presence of polymorphism adds another restriction site to the 266 bp fragment, which then leads to presentation of three degradation products: 171, 50 and 45 bp.

### Statistical analysis

The chi-square and Student t tests were used for baseline comparisons. Each GP mutation was divided into two genotype groups for bivariate analysis (homozygous wild versus heterozygous and homozygous mutants). The Spearman test was used for correlations between GP mutations and platelet inhibition data. Using in-hospital death as a dependent variable, we performed logistic regression that included independent variables with P < 0.25. The significance level used for the statistical tests was 5% (P < 0.05). Statistical analyses were performed using the SAS for Windows software (Statistical Analysis System), version 9.2 (SAS Institute Inc., 1989-1996; Cary, NC, USA).

## RESULTS

The patients’ mean age was 61.8 ± 11.7 years and 30 (60%) were men. Hypertension, dyslipidemia, previous coronary artery disease and diabetes were highly prevalent among the patients and occurred, respectively, in 47 (94%), 39 (78%), 27 (54%) and 25 (50%) of the patients. The prevalence of diabetes was 25%, and 24% were active smokers. Previous stroke was reported by 10% of the patients. In-hospital death occurred in the cases of seven patients (14%): six (12%) due to cardiogenic shock, and one (2%) due to septicemia. The distributions of these data according to the genotype group (homozygous wild versus heterozygous and homozygous mutant) of each GP mutation are shown in Table 2. The prevalences of hypertension, dyslipidemia, diabetes, smoking, previous coronary artery disease, previous stroke and all causes of death were similar between the groups. Platelet aggregations before tirofiban administration were also similar between the GPIa, Ib and IIb mutation groups, but not for the GPIIIa group. The GPIIIa mutation had lower baseline aggregation than the wild genotype (41.0% ± 22.1% versus 55.9% ± 20.8%; P = 0.035). A moderate correlation was observed between GPIIIa mutation and baseline platelet aggregation (r = -0.31; P = 0.032). After tirofiban administration, platelet GPIa, Ib, IIb and IIIa mutations did not alter inhibition of platelet aggregation or all causes of death (Table 2). Multivariate logistic regression did not reveal any GP mutation that was an independent variable for in-hospital death (Table 3).

**Table 2.**
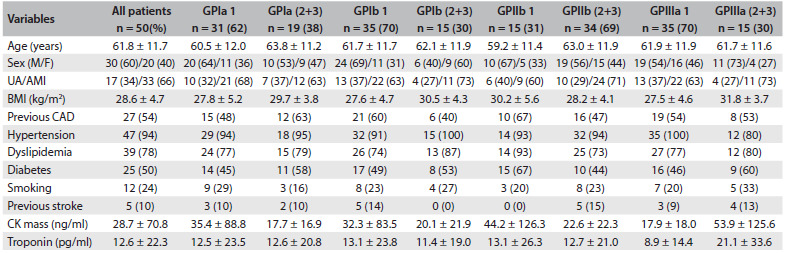
Demographic variables and laboratory data relating to 50 patients with unstable angina and non-ST-segment elevation myocardial infarction

GP = glycoprotein; 1 = homozygous wild; 2 = heterozygous; 3 = homozygous mutant; UA = unstable angina; AMI = acute myocardial infarction; M = male; F = female; CAD = coronary artery disease; CK = creatine kinase. Value in parentheses are percentages.

**Table 3.**
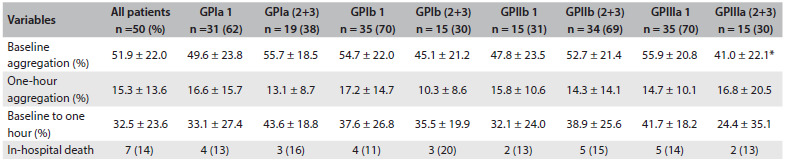
Aggregation data and incidence of death among 50 patients with unstable angina and non-ST-segment elevation myocardial infarction

*P = 0.035; GP = glycoprotein; 1 = homozygous wild; 2 = heterozygous; 3 = homozygous mutant; one-hour aggregation and inhibition (%) refer to the percentage aggregation or inhibition one hour after tirofiban administration. Value in parentheses are percentages.

## DISCUSSION

The four polymorphisms of platelet glycoprotein analyzed in this study did not have any influence on the level of platelet inhibition in response to a standard dose of tirofiban, a specific inhibitor of GPIIbIIIa, in patients with acute coronary syndrome. The mutations analyzed were selected because these mutations present high frequency in our population.^11^ The patients were treated in accordance with the guidelines of the American Heart Association for patients with unstable angina or NSTEMI.^5^ Aspirin was administered at least three hours before introduction of tirofiban. Blood samples were obtained before and one hour after tirofiban administration, for analysis on platelet aggregation in response to tirofiban.

### Prior tirofiban administration

There was a lower degree of inhibition of platelet aggregation with aspirin among the patients with the glycoprotein IIIa PLA2 mutation, before administration of tirofiban. This mutation was most often associated with increased resistance to inhibition among the platelet polymorphisms analyzed in this study.^18^ Szczeklik et al. assessed the association between genotypes with PlA2 mutation and platelet aggregation in response to aspirin, in 80 healthy subjects. Individuals carrying the mutation PlA2 were associated with greater resistance of platelet aggregation to aspirin.^19^ Among 82 patients on aspirin and clopidogrel, Angiolillo et al. showed that there was higher platelet aggregation in response to the agonists collagen, epinephrine and ADP, in patients with the GPIa C807T mutation.^20^ Furthermore, Gonzalez-Conejero et al. did not observe any association between the mutations GPIIbIIIa PlA2 and GPIaIIa C807T and the efficacy of aspirin for platelet inhibition.^21^ The effectiveness of platelet inhibition was higher among subjects treated with higher doses of aspirin. These authors concluded that aspirin resistance was unlikely, but when it occurred, it would probably be dose-dependent and not influenced by mutations of GPIIbIIIa and GPIa/IIa.

The association between mutations of GPIIbIIIa and GPIa/IIa and higher resistance of platelet inhibition to aspirin and clopidogrel is very questionable. Recent systematic reviews and meta-analyses by Floyd et al. showed conflicting associations between the GPIIIa PlA2 allele and resistance to antiplatelets and to cardiovascular diseases.^22^ In these studies, the authors found an association between PlA2 mutation and ischemic stroke; however, no association was observed between the mutation and resistance to platelet inhibition with aspirin and clopidogrel, either in healthy subjects or in patients with cardiovascular diseases.^23^

### After tirofiban administration

Our study showed that mutations of the glycoproteins analyzed did not influence platelet aggregation one hour after administration of tirofiban. However, it can be argued that inhibition of platelet aggregation using tirofiban is less effective than that of other GPIIbIIIa inhibitors.^5^^,^^6^^,^^24^ This lower efficacy could be related to the genetic variability of the glycoproteins involved in thrombus formation.

Nevertheless, the results from studies on the influence of genetic polymorphisms of platelet glycoproteins towards lower platelet response to GPIIbIIIa inhibitors have been contradictory. O’Connor et al. showed that there was higher incidence of major coronary events among patients with acute coronary syndrome with GPIIIa PlA2 mutation who were treated with orbofiban, an oral GPIIbIIIa antagonist, thus signaling that there might be an association between this mutation and coronary thrombosis.^25^ Wheeler et al. showed that there was less platelet inhibition through using abciximab in patients with the PlA2 mutation who underwent percutaneous coronary intervention.^26^ Weber et al. conducted an *in vitro* study on the antiplatelet effects of three GPIIbIIIa inhibitors (abciximab, tirofiban and eptifibatide) among healthy individuals and patients with stable coronary artery disease.^27^ They confirmed that there was great variability of the inhibitory response to GPIIbIIIa inhibitors, but that this variability was not associated with GPIIbIIIa PlA2 mutation. Verdoia et al. analyzed the platelet response to GPIIbIIIa inhibitors among 80 patients undergoing percutaneous coronary revascularization (40 patients with abciximab; 40 patients with eptifibatide or tirofiban).^28^ Aggregation tests were performed at baseline and after 10 min, 1 h and 4 h after GPIIbIIIa inhibitor administration. PlA2 mutation was present in 26 patients (32.5%). The clinical and angiographic features were similar between groups of carriers and noncarriers of the PLA2 mutation, except with regard to in-stent restenosis, which was more frequent among patients with PlA2. The PlA2 mutation did not affect the platelet response to GPIIbIIIa inhibitors.

Although the initial studies signaled that genetic polymorphisms of platelet glycoproteins had an influence on platelet response, this interaction was not observed in our study or proven by other more recent studies.^25^^,^^26^^,^^27^^,^^28^ However, variability of platelet response to GPIIbIIIa inhibitors exists and can be correlated with other factors, such as drug dose-dependence, the method used to assess platelet aggregation, pharmacodynamics, concomitant use of dual antiplatelet aggregation, rheological characteristics of blood coagulation and other such things. Khaspekova et al. analyzed the platelet response among patients with acute coronary syndrome and found that the expression of platelet GPIb and GPIIbIIIa correlated with the average volume of platelets and not the genetic polymorphism of the GPIIIa Leu33Pro and GPIbα Thr145Met mutations.^29^ Schneider et al. showed that the lesser degree of initial inhibition of platelet aggregation using tirofiban was dose-dependent compared to abciximab.”^30^ A recent meta-analysis showed that there was a reduction in major adverse coronary events through addition of tirofiban to aspirin and clopidogrel, among patients with ST-elevation myocardial infarction prior to percutaneous coronary intervention.^31^ Blood rheological changes, such as blood viscosity, increased serum levels of fibrinogen, inflammatory cytokines and others, could explain the variability of platelet response. It is well known that these factors favor thrombotic process by acting at various levels of the coagulation system.^32^

## CONCLUSION

In conclusion, this study showed that the GPIa, Ib, IIb and IIIa mutations analyzed did not influence platelet aggregation in response to tirofiban in patients with acute coronary syndrome. This result suggests that, despite the possibility that interindividual differences in platelet response to GPIIbIIIa inhibitors might have a genetic component, this probably depends on deeper interaction between genes and not just the interaction of a few single glycoprotein mutations. The small number of patients, homozygous and heterozygous mutant genotype groupings and influence of prior use of aspirin may be important limitations of this study. Similarly, it may not be possible to generalize our findings to other antiplatelet drugs.
